# The oncogenic role of the cochaperone Sgt1

**DOI:** 10.1038/oncsis.2015.12

**Published:** 2015-05-18

**Authors:** H Ogi, Y Sakuraba, R Kitagawa, L Xiao, C Shen, M A Cynthia, S Ohta, M A Arnold, N Ramirez, P J Houghton, K Kitagawa

**Affiliations:** 1 Center for Childhood Cancer and Blood Diseases, The Research Institute at Nationwide Children's Hospital, Columbus, OH, USA; 2 Department of Pathology and Laboratory Medicine, The Research Institute at Nationwide Children's Hospital, Columbus, OH, USA; 3 Department of Pediatrics, College of Medicine, The Ohio State University, Columbus, OH, USA; 4 Children's Cancer Research Institute, San Antonio, TX, USA

## Abstract

Sgt1/Sugt1, a cochaperone of Hsp90, is involved in several cellular activities including Cullin E3 ubiqutin ligase activity. The high level of Sgt1 expression in colorectal and gastric tumors suggests that Sgt1 is involved in tumorigenesis. Here, we report that Sgt1 is overexpressed in colon, breast and lung tumor tissues and in Ewing sarcoma and rhabdomyosarcoma xenografts. We also found that *Sgt1* heterozygous knockout resulted in suppressed Hras-mediated transformation *in vitro* and tumor formation in *p53*^−/−^ mouse embryonic fibroblast cells and significantly increased survival of *p53*^−/−^ mice. Moreover, depletion of Sgt1 inhibited the growth of Ewing sarcoma and rhabdomyosarcoma cells and destabilized EWS-FLI1 and PAX3-FOXO1 oncogenic fusion proteins, respectively, which are required for cellular growth. Our results suggest that Sgt1 contributes to cancer development by stabilizing oncoproteins and that Sgt1 is a potential therapeutic target.

## Introduction

Dysregulation of oncogenes and tumor suppressor genes is a common feature of different types of cancer. Because aberrant oncoproteins are unstable, cancer cells utilize Hsp90 as a chaperone to promote folding and function of mutated or overexpressed oncoproteins.^1^ The proliferation and survival of cancer cells can often be suppressed by the inhibition of one or more oncoproteins.^2^ Therefore, Hsp90 inhibitors have been developed and are under investigation as cancer therapy in clinical trials.^1^

Sgt1/Sugt1 (suppressor of G2 allele of *skp1*) is a highly conserved protein that functions as a cochaperone of Hsp90.^3, 4, 5, 6, 7^ Cochaperones, which interact with and are required for Hsp90 function, regulate the ATPase activity of Hsp90, recruit client proteins to Hsp90 and have been proposed as potential targets for cancer therapy.^1^ Presumably as a cochaperone, Sgt1 is involved in several specific cellular functions including ubiquitination,^8^ cyclic AMP pathway,^9, 10^ centrosome maturation,^11^ kinetochore assembly^8, 12^ and immune response.^13, 14, 15, 16^ Sgt1 and Hsp90 are required for kinetochore assembly because of their enhancement of the localization of kinetochore proteins.^12, 17, 18^ In addition, Sgt1 and Hsp90 participate in kinetochore-microtuble attachment by stabilizing the Mis12 complex at kinetochores.^17, 19^ Therefore, knockdown of Sgt1 expression induces misalignment of chromosomes, activation of a weakened spindle checkpoint and, possibly, the occurrence of aneuploidy.^12, 17, 18, 19^ In addition to these functions, Sgt1 and Hsp90 play a role in neuroblast cortical polarity through localization of Par and Pins complexes^20^ and in HGF-mediated epithelial morphogenesis through stabilization of Scribble.^21^

Overexpression of Sgt1 in tumor tissues has been reported. *Sgt1* mRNA levels are elevated in colorectal cancer, and this increased expression is linked to an increased rate of recurrence and poorer prognosis.^22^ In gastric tumor cells, overexpression of Sgt1 upregulates Akt phosphorylation through the degradation of the phosphatase PHLPP1 by enhancing the interaction of PHLPP1 with SCF-β-TrCP.^23^ These findings suggest that overexpression of Sgt1 is involved in tumorigenesis. However, the function of Sgt1 in cancer development remains obscure.

Here, we report the findings of our investigation of Sgt1 protein levels in tumor tissues and pediatric tumor xenografts. Sgt1 was highly expressed in colon, breast and lung tumor tissues and in Ewing sarcoma and rhabdomyosarcoma xenografts. Reduction of Sgt1 altered the ability of mouse embryonic fibroblast (MEF) cells to undergo transformation and form tumors and the Sgt1 reduction resulted in increased survival of *p53* knockout mice. These effects appeared to be independent of mitotic defects. Knockdown of Sgt1 expression inhibited the proliferation of cancer cells and destabilized oncoproteins that are required for the growth of Ewing sarcoma and rhabdomyosarcoma cells. Our results suggest that Sgt1 is involved in cancer development, possibly by stabilizing oncoproteins, and highlight Sgt1 as a potential therapeutic target.

## Results

### Sgt1 protein levels are elevated in tumor tissues and in Ewing sarcoma and rhabdomyosarcoma xenografts

**Figure 1:**
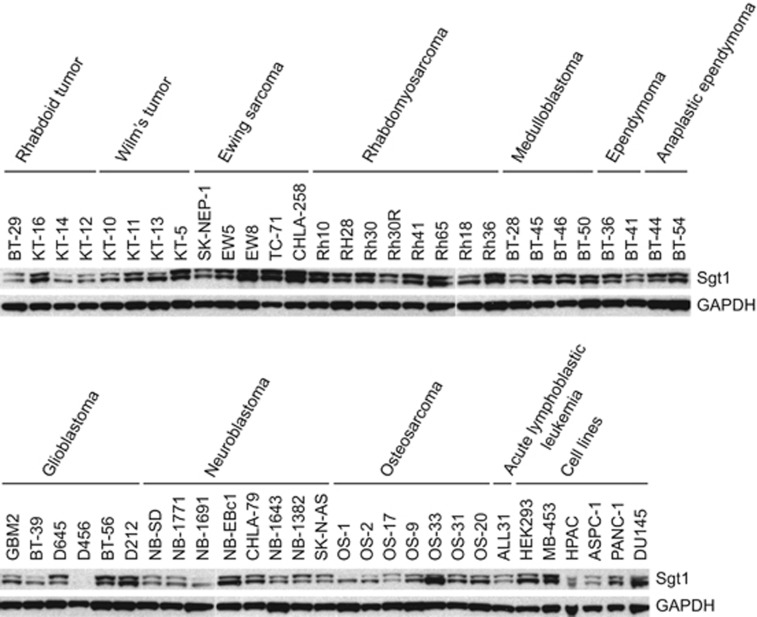
Sgt1 expression in pediatric solid tumor xenografts. Protein extracts of 50 solid tumor xenografts obtained from the PPTP, the ALL31 xenograft, and the cultured cells HEK293, MB-453, HPAC, ASPC-1, PANC-1 and DU145 were used to detect Sgt1 and GAPDH by immunoblotting.

To check protein levels of Sgt1 in tumor tissues, we performed immunoblotting analysis of tumor tissues (colon adenocarcinoma, breast ductal carcinoma, lung adenocarcinoma and lung squamous cell carcinoma) and normal adjacent tissues from the same patient. Levels of Sgt1 protein were greater in most tumor tissues than in normal adjacent tissues (Supplementary Figure S1). In additional immunoblotting studies, we also evaluated Sgt1 protein levels in 50 solid tumor xenografts obtained from the Pediatric Preclinical Testing Program (PPTP). Overexpression of Sgt1 protein was observed in xenografts, especially those of Ewing sarcoma and rhabdomyosarcoma (Figure 1).

Furthermore, the expression of Sgt1 protein was evaluated by immunohistochemistry of resected breast carcinoma, lung carcinoma, Ewing sarcoma and rhabdomyosarcoma specimens using an anti-Sgt1 antibody. Sgt1 appears to be more abundant in cancer tissues than in normal tissues (Supplementary Figure S5).

These results indicate that the Sgt1 protein is overexpressed in a broad range of tumor tissues.

### *Sgt1* heterozygous knockout decreases transformation and tumorigenicity of MEF cells

To investigate the role of Sgt1 in tumorigenesis, we first produced *Sgt1* knockout mice by using the BayGenomics ES cell line RRS405. The gene trap vector was inserted into intron 2 of the *Sgt1* gene (Supplementary Figure S2A), and this insertion resulted in the expression of a truncated Sgt1 protein fused with a β-geo marker. We crossed *Sgt1* heterozygous knockout female and male mice; however, *Sgt1* homozygous knockout mice were not obtained (Supplementary Figure S2B). *Sgt1* homozygous knockout embryos at embryonic day (E) 3.5 were obtained by *in vitro* fertilization, whereas *Sgt1* homozygous knockout embryos from E8.5 to E14.5 were not obtained (Supplementary Figure S2C). These results indicate that the truncated Sgt1 protein is not functional and that *Sgt1* homozygous knockout results in early embryonic lethality in mice.

**Figure 2:**
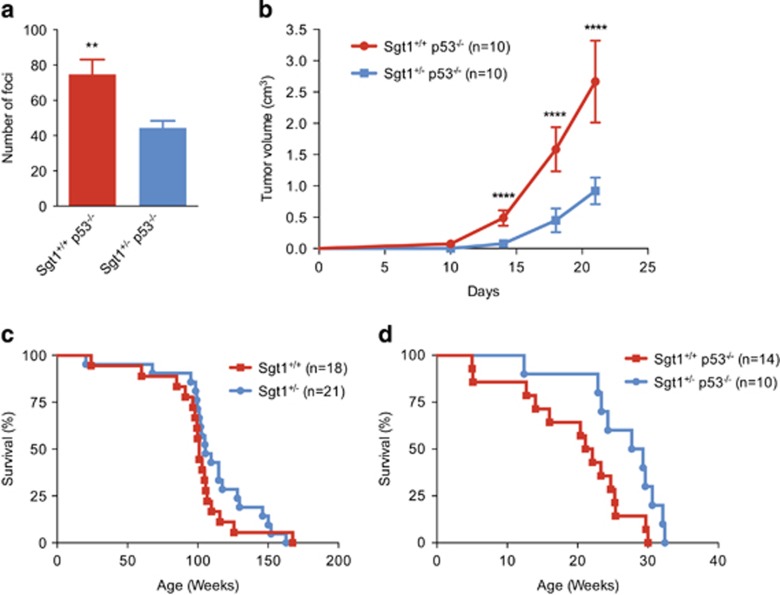
*Sgt1* heterozygous knockout suppresses Hras-mediated transformation and tumorigenicity of *p53*^−/−^ MEF cells and increases survival of *p53*^−/−^ mice. (**a**) *Sgt1*^+/+^ *p53*^−/−^ and *Sgt1*^+/−^ *p53*^−/−^ MEF cells transduced with Hras were used for focus-formation assays. After 2 weeks of incubation, cells were stained and the number of foci was counted. The experiments were repeated three times, and similar results were obtained. Average values±s.d. are shown (∗∗*P*<0.01; unpaired *t* test). (**b**) Hras-transduced MEF cells were injected into immunodeficient CB17SC-F *scid*^−/−^ female mice. Tumor volumes were measured at indicated time points. Average values±s.d. are shown (∗∗∗∗*P*<0.0001; unpaired *t*-test). Mice in the blue curve were killed owing to dehydration on the twenty-first day or the twenty-fourth day. (**c**) The survival estimates of *Sgt1*^+/+^ and *Sgt1*^+/−^ mice are shown. (**d**) The survival curve of *Sgt1*^+/+^ *p53*^−/−^ and *Sgt1*^+/−^ *p53*^−/−^ mice are shown (*P*<0.05; log-rank test).

To evaluate the effect of decreased Sgt1 protein, we established *Sgt1*^+/+^ *p53*^−/−^ and *Sgt1*^+/−^ *p53*^−/−^ MEF cells by crossing *Sgt1* knockout and *p53* knockout mice. Hras was expressed in these MEF cells as a result of retrovirus transduction, and focus formation assays were performed. Both types of cells that expressed Hras formed foci; however, the number of foci formed by *Sgt1*^+/−^ *p53*^−/−^ MEF cells was significantly less than that formed by *Sgt1*^+/+^ *p53*^−/−^ MEF cells (Figure 2a). No proliferation defects in *Sgt1*^+/−^ *p53*^−/−^ MEF cells were observed (data not shown). To test the effect of Sgt1 reduction on tumorigenicity, we conducted an allograft experiment in which Hras-transduced *Sgt1*^+/+^ *p53*^−/−^ and *Sgt1*^+/−^ *p53*^−/−^ MEF cells were injected into immunodeficient mice and tumor formation was monitored. *In vivo* tumor formation of Hras-transduced *Sgt1*^+/−^ *p53*^−/−^ MEF cells was significantly slower than that of Hras-transduced *Sgt1*^+/+^ *p53*^−/−^ MEF cells (Figure 2b).

### *Sgt1* heterozygous knockout increases the survival of *p53*^−/−^ mice

To test the effect of *Sgt1* heterozygous knockout *in vivo*, the survival of *Sgt1*^+/+^ and *Sgt1*^+/−^ mice was monitored. No significant difference in the survival between *Sgt1*^+/+^ and *Sgt1*^+/−^ mice was observed (Figure 2c). *Sgt1*^+/−^ mice and *Sgt1*^+/−^ MEF cells did not show any obvious mutant phenotypes (data not shown), a result that indicated that one copy of *Sgt1* is sufficient for viability.

To analyze the *in vivo* effect of the *Sgt1* heterozygous knockout in mice that are deficient in a tumor suppressor gene, *Sgt1*^+/+^ *p53*^−/−^ and *Sgt1*^+/−^ *p53*^−/−^ mice were produced, and the survival of these mice were monitored. *Sgt1* heterozygous knockout resulted in significantly longer survival of *p53*^−/−^ mice than *Sgt1* wild-type mice (Figure 2d).

Taken together, our results indicate that *Sgt1* reduction suppresses Hras-mediated transformation and tumorigenicity of *p53*^−/−^ MEF cells, and this suppression may result in the prolonged survival of *p53*^−/−^ mice.

### Kinetochore formation, spindle checkpoint and ploidy are normal in *Sgt1*^+/−^ MEF cells

Aneuploidy (that is, the state of having an abnormal number of chromosomes) that results from *CENP-E* (a kinetochore motor protein) heterozygous knockout appears to inhibit tumorigenesis in *p19*/*ARF*^−/−^ mice.^24, 25^ *Sgt1* depletion by short interfering RNA (siRNA) in HeLa cells causes delocalization of kinetochore proteins and activation of the weakened spindle checkpoint, which may result in aneuploidy.^12, 17, 19^ Therefore, we hypothesized that aneuploidy is the mechanism of tumor suppression in *Sgt1* heterozygous knockout mice. Our phenotype analysis of MEF cells found that the level of Sgt1 protein in *Sgt1*^+/−^ MEF cells was reduced to about 30% of the level in *Sgt1*^+/+^ MEF cells (Supplementary Figure S3A). Indirect immunofluorescence microscopy of kinetochore localization showed that CENP-H signals at kinetochores in *Sgt1*^+/−^ MEF cells were indistinguishable from those in *Sgt1*^+/+^ MEF cells (Supplementary Figure S3B). Also, the mitotic index in response to paclitaxel was indistinguishable between *Sgt1*^+/+^ *p53*^−/−^ MEF cells and *Sgt1*^+/−^ *p53*^−/−^ MEF cells (Supplementary Figure S4A). Furthermore, chromosome numbers were similar between *Sgt1*^+/+^ MEF cells and *Sgt1*^+/−^ MEF cells (Supplementary Figure S3C). These results indicate that kinetochores are assembled properly and that the spindle checkpoint is normal in *Sgt1*^+/−^ MEF cells and that there is no difference in ploidy between *Sgt1*^+/+^ MEF cells and *Sgt1*^+/−^ MEF cells. Therefore, we concluded that aneuploidy does not seem to be the mechanism of the tumor suppression in *Sgt1*^+/−^ mice.

### *Sgt1* heterozygous knockout does not increase senescent and apoptotic cells

Cellular senescence and apoptosis are major mechanisms of tumor suppression.^26^ Cellular senescence was observed in *Skp2*-deficient MEF cells in an *ARF*-*p53-*independent manner.^27^ Sgt1 interacts with Skp1,^8, 18^ which interacts with Skp2.^28^ Our analysis found no significant difference in HRas-induced senescence and apoptosis between *Sgt1*^+/−^ *p53*^−/−^ MEF cells and *Sgt1*^+/+^ *p53*^−/−^ MEF cells (Supplementary Figures S4B and S4C). These results suggest that senescence and apoptosis are not the mechanism of tumor suppression.

### Knockdown of *Sgt1* expression inhibits the proliferation of cancer cells

**Figure 3:**
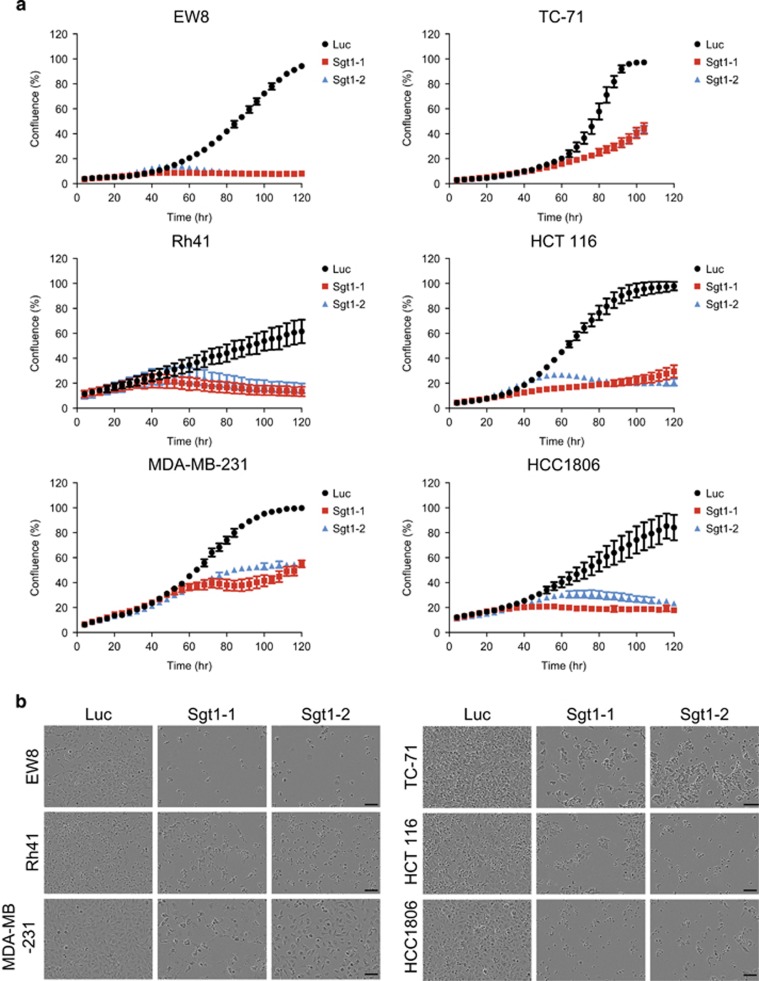
Knockdown of Sgt1 expression inhibits the growth of cancer cells. (**a**) EW8, TC-71, Rh41, HCT 116, MDA-MB-231 and HCC1806 cells were transfected with luciferase siRNA (Luc) and two independent *Sgt1* siRNAs (*Sgt1-1* and *Sgt1-2*). Proliferation of the cells was monitored at 4-h intervals, and data were analyzed by IncuCyte software. The experiments were repeated three times, and similar results were obtained. Average values±s.d. are shown. (**b**) Representative images of cells transfected with siRNAs are shown. Bars represent a distance of 100 μm.

**Figure 4:**
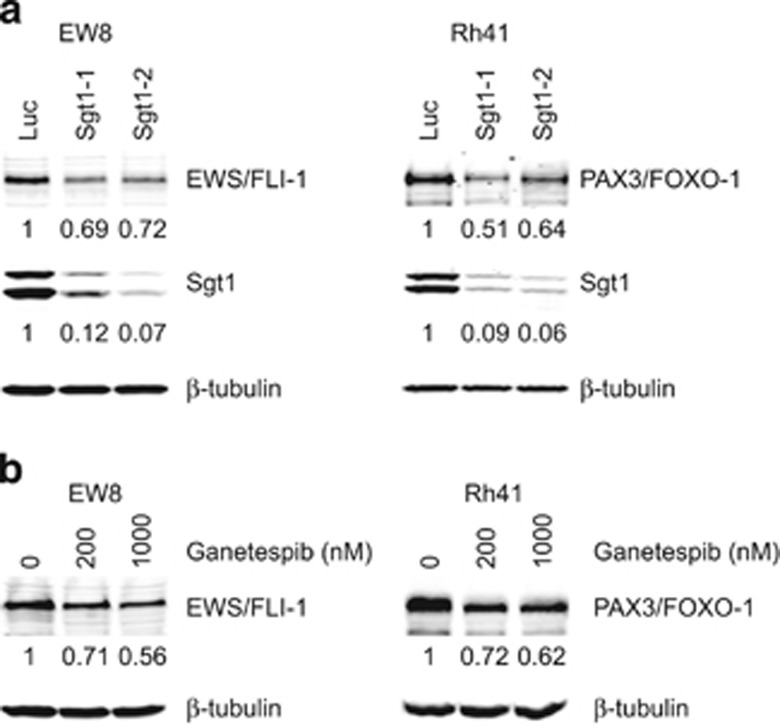
EWS-FLI1 and PAX3-FOXO1 oncoproteins were destabilized after knockdown of Sgt1 expression or inhibition of Hsp90. (**a** and **b**) Indicated proteins in EW8 or Rh41 were detected by immunoblotting at 72 h after siRNA treatment (**a**) or at 24 h after ganetespib treatment (**b**). Levels of EWS-FLI1, PAX3-FOXO1 and Sgt1 were normalized to β-tubulin, and the protein level of control cells was established as a value of 1.

Because the reduced expression of Sgt1 protein that resulted from *Sgt1* heterozygous knockout suppressed Hras-mediated transformation and tumorigenicity of *p53*^−/−^ MEF cells and prolonged the survival of *p53*^−/−^ mice (Figure 2), we hypothesized that Sgt1 is a potential target for cancer therapy. To test this hypothesis, we examined whether depletion of Sgt1 affects the proliferation of *Sgt1*-overexpressing cancer cells (that is, Ewing sarcoma cell lines EW8 and TC-71 and rhabdomyosarcoma cell line Rh41). To knock down *Sgt1* expression, we incubated cancer cells transfected with two independent siRNAs against *Sgt1*^12, 18^ (Figure 4a and Supplementary Figure S4D) and recorded the extent of cell growth at 4-h intervals. Knockdown of *Sgt1* expression significantly inhibited the proliferation of cells when compared with that of cells subjected to a luciferase control (Figure 3).

### Sgt1 and Hsp90 stabilize the oncoproteins EWS-FLI1 and PAX3-FOXO1

Sgt1 interacts with Hsp90 and functions as a cochaperone of Hsp90.^3, 6, 11, 17, 21^ Sgt1 is involved in stability of Polo, Mis12 complex and Scribble that are required for centrosome maturation, proper kinetochore assembly or HGF-mediated epithelial morphogenesis, respectively.^11, 17, 21^ Therefore, we hypothesized that Sgt1 may be involved in stability of the oncofusion proteins that are essential for the proliferation of cancer cells. To test this hypothesis, we evaluated the levels of the oncogenic fusion proteins EWS-FLI1 and PAX3-FOXO1, which are required for the proliferation of Ewing sarcoma and alveolar rhabdomyosarcoma cell lines, respectively.^29, 30^ Protein levels of EWS-FLI in EW8 cells and PAX3-FOXO1 in Rh41 cells were reduced by *Sgt1* depletion or ganetespib (an Hsp90 inhibitor) treatment (Figures 4a and b).

These may imply that Sgt1 and Hsp90 are required for the proliferation of cancer cells by stabilizing proteins essential to that growth.

## Discussion

In our current study and in previous reports, overexpression of Sgt1 in tumor tissues and xenografts was observed.^22, 23^ *Sgt1* heterozygous knockout decreased Hras-mediated transformation and tumorigenicity of *p53*^−/−^ MEF cells and extended the survival period of *p53*^−/−^ mice. These results prompted us to hypothesize that *Sgt1* is an oncogene. To test this hypothesis, we conducted focus-formation assays using *p53*^−/−^ and *ARF*^−/−^ MEF cells that overexpressed mouse *Sgt1* and wild-type MEF cells that coexpressed *Sgt1* and the immortalizing oncogene c-*Myc*. However, the results under these conditions were negative (data not shown). Next, we tested the effect of overexpression of *Sgt1* on Hras-mediated transformation of MEF cells in focus-formation assays. Overexpression of *Sgt1* and *Hras* in *p53*^−/−^ MEF cells had no effect on Hras-mediated transformation (data not shown). These findings suggest that *Sgt1* may not be an authentic oncogene. Like *Sgt1*, *Cdc37* is a co-chaperone of Hsp90, is overexpressed in different types of cancer cells, and is thought to be an oncogene.^31^ Although overexpression of *Cdc37* in mice causes tumors after long latency,^32^ there has been no report that describes cellular transformation induced by *Cdc37* overexpression. It will be interesting to test whether overexpression of *Sgt1* affects the long latency of tumors in *Sgt1* transgenic mice.

What is the molecular mechanism of tumor suppression caused by the *Sgt1* heterozygous knockout? The results of experiments described in this article indicate that mitotic defects, senescence, and apoptosis are not the mechanisms. Recently, Gao *et al.*^23^ reported that overexpression of Sgt1 upregulates Akt phosphorylation through enhancement of SCF-β-TrCP-dependent degradation of the phosphatase PHLPP1 in gastric cancer cells. However, we found no difference in Akt phosphorylation and PHLPP1 protein levels among *p53*^−/−^ *Sgt1*^+/−^, *p53*^−/−^ *Sgt1*^+/+^ or Sgt1-overexpressing MEF cells (data not shown). In light of our finding that levels of the EWS-FLI1 protein in EW8 cells or PAX3-FOXO1 protein in Rh41 cells were decreased by the knockdown of *Sgt1* expression, it is plausible that destabilization of the onco-proteins is a mechanism of tumor suppression that results from the *Sgt1* heterozygous knockout. Such a destabilization mechanism functions in Sgt1-depleted cancer cells.

An Hsp90 cochaperone that is overexpressed in cancers is a potential target for cancer therapy, because Hsp90 requires cochaperones to function.^33^ Knockdown of *Cdc37* expression inhibits the growth of cancer cells and xenografts.^34^ Given that Sgt1 is a cochaperone of Hsp90, that Sgt1 is overexpressed in tumor cells and that decreased Sgt1 protein suppresses cellular transformation and allograft growth, we believe that Sgt1 could be a potential target for cancer therapy. Consistent with this possibility is the previous finding that knockdown of *Sgt1* expression inhibits the growth of cancer cells.^23^ Sgt1 plays a role in various cellular functions by stabilizing the proteins together with Hsp90. A mutation in *Sgt1* decreases levels of Polo.^11^ Knockdown of *Sgt1* or *Hsp90* expression or inhibition of Hsp90 by 17-AAG decreases levels of the Mis12 complex^17^ or Scribble.^21^ Reduction of these proteins causes defects in centrosome maturation, kinetochore formation and epithelial morphogenesis. Therefore, it is possible that Sgt1 is involved in cancer development by stabilizing proteins that are required for the growth of cancer cells. The oncogenic fusion proteins EWS-FLI1 and PAX3-FOXO1 are required for the growth of Ewing sarcoma and rhabdomyosarcoma cells, respectively.^29, 30^ Levels of the EWS-FLI1 protein in EW8 cells or the PAX3-FOXO1 protein in Rh41 cells were decreased by knockdown of *Sgt1* expression. These results corroborate the potential of Sgt1 for cancer therapy. Further study is needed to identify client proteins of Sgt1 in other types of cancer cells.

## Materials and methods

### Reagents

The following antibodies were used in our experiments: mouse anti-Sgt1 (BD Biosciences, San Jose, CA, USA), rabbit anti-GAPDH (Abcam, Cambridge, MA, USA), rabbit anti-Fli1, rabbit anti-FKHR (Santa Cruz Biotechnology, Dallas, TX, USA), mouse anti-β-tubulin (MP Biomedicals, Santa Ana, CA, USA), mouse anti-CENP-H (BD Biosciences), human anti-centromere autoimmune serum (a generous gift from Dr. William R. Brinkley, Baylor College of Medicine), rabbit anti-phospho-histone H3 (Ser10) (EMD Millipore, Billerica, MA, USA), goat anti-mouse IgG-HRP, goat anti-rabbit IgG-HRP (Santa Cruz Biotechnology), IRDye 680RD goat anti-mouse IgG (H+L), IRDye 800CW goat anti-mouse IgG (H+L), IRDye 800CW goat anti-rabbit IgG (H+L) (LI-COR), Alexa Fluor 488 goat anti-mouse IgG (H+L), Alexa Fluor 488 goat anti-rabbit IgG (H+L) and Alexa Fluor 594 goat anti-human IgG (H+L) (Life Technologies, Grand Island, NY, USA).

The ready-to-use membranes purchased from Novus Biologicals (Littleton, CO, USA) were INSTA-Blot Male Lung Tissue Oncopair (catalog number IMB-127a), INSTA-Blot Female Lung Tissue Oncopair (catalog number IMB-128a), INSTA-Blot Breast Tissue Oncopair (catalog number IMB-130a), INSTA-Blot Breast Tissue Oncopair (catalog number IMB-130e) and INSTA-Blot Colon Tissue Oncopair (catalog number IMB-131a).

Immunoblotting was performed by standard procedure, and immunolabeled proteins were detected by ECL Plus Western Blotting Detection Reagents (GE-Healthcare Life Sciences, Pittsburgh, PA, USA) or ODYSSEY CLx (LI-COR, Lincoln, NE, USA).

The siRNAs against luciferase and Sgt1 were previously described.^11, 18^ Luciferase siRNA (5′-CUUACGCUGAGUACUUCGATT), Sgt1-1 siRNA (5′-GCUAGAGGGGCAAGGAGAUTT) and Sgt1-2 siRNA (5′-AAGGCUUUGGAACAGAAACCA) were synthesized by the Hartwell Center for Bioinformatics and Biotechnology at St. Jude Children's Research Hospital.

### Cell lines, primary cells and cell culture

HCT 116, MDA-MB-231 and HCC1806 cells were obtained from American Type Culture Collection (ATCC, Manassas, VA, USA). EW8, Rh41 and HCC1806 cells were maintained in RPMI 1640 (Lonza, Portsmouth, NH, USA) supplemented with 10% fetal bovine serum (FBS; Life Technologies), TC-71 was maintained in IMDM (Lonza) supplemented with 10% FBS and ITS (Life Technologies), HCT 116 cells were maintained in McCoy's 5A (ATCC) supplemented with 10% FBS, and MDA-MB-231 cells were maintained in DMEM (Lonza) supplemented with 10% FBS at 37 °C with 5% CO_2_.

Primary MEF cells were derived from E13.5 or E14.5 embryos. The *p53*^−/−^ and *ARF*^−/−^ MEF cells are generous gifts from Dr. Martine F. Roussel (St. Jude Children's Research Hospital). MEF cells were maintained in DMEM (Lonza) that contained 10% FBS, 1 × MEM non-essential amino acid, 55 μM 2-mercaptoethanol and 10 μg/ml gentamicin (Life Technologies) at 37 °C with 5% CO_2_. Early passage (P2-P5) MEF cells were used for the experiments. Exceptions were *p53*^−/−^ and *ARF*^−/−^ MEF cells.

### Retrovirus production and focus-formation assay

Plat-E cells (Cell Biolabs, San Diego, CA, USA) were used to produce retrovirus according to the manufacturer's instructions. Plat-E cells were transfected with pBABE-puro and pBABE-puro Hras V12 (a generous gift from Martine F. Roussel/Scott Lowe) by using FuGENE 6 (Promega, Fitchburg, WI, USA). Retroviral supernatant was filtered and mixed with 4 μg/ml polybrene (EMD Millipore). MEF cells were infected with retroviral supernatant twice and selected in the presence of 2 μg/ml of puromycin (Sigma-Aldrich, St. Louis, MO, USA) for 4 days.

Focus-formation assays were performed as previously described.^35^ Briefly, 10^3^ MEF cells infected with retrovirus were mixed with 3 × 10^5^ uninfected MEF cells, and the mixture was cultured in a 100-mm dish in triplicate. Medium was changed every 2 or 3 days. After 2 weeks incubation, foci were stained with giemsa stain (Sigma-Aldrich), and the number of foci was counted.

### Mouse strains

BayGenomics ES cell line RRS405 (129P2/OlaHsd) was purchased from Mutant Mouse Regional Resource Centers (http://www.mmrrc.org/index.php). This ES cell line was used to produce *Sgt1* knockout mice by standard procedures at the St. Jude Transgenic Core Facility. The *p53* knockout mice (C57BL/6J) were purchased from The Jackson Laboratory (stock number 002101) (Bar Harbor, ME, USA). All mice have mixed 129P2/OlaHsd and C57BL/6J genetic background. All mice, including those used in xenograft and allograft experiments, were maintained under barrier conditions, and experiments were conducted according to protocols and conditions approved by the institutional animal care and use committee.

### Solid tumor xenograft and allograft

Immunodeficient CB17SC-F *scid*^−/−^ female mice (Taconic Farms, Germantown, NY, USA)^36^ were used to propagate subcutaneously implanted tumors. Tumors were excised, rapidly frozen in liquid nitrogen and pulverized under liquid nitrogen. Total proteins were extracted with cell lysis buffer (Cell Signaling Technology, Danvers, MA, USA) supplemented with protease inhibitors and protein phosphatase inhibitors (Roche, Indianapolis, IN, USA). Thirty-five micrograms of total proteins were used in immunoblotting experiments to detect Sgt1 and GAPDH.

The tumorigenic potential of MEF cells was evaluated by injecting 10^6^ cells (volume, 0.1 ml) into the left flank of immunodeficient CB17SC-F *scid*^*−*/*−*^ female mice. Tumor volume (cm^3^) was measured with calipers and determined as previously described.^37^ Tumor volume was measured biweekly at the initial observation of the tumor growth until endpoint criteria were achieved. Tumor volume was calculated according to the following formula: (π/6) · *d*^3^, where *d* represents the mean diameter.^37^ Mice were humanely killed at the endpoint. The endpoint criteria met by this study were follows: a tumor volume of 2.24 cm^3^, ulceration of the tumor and dehydration or poor condition of the animal.

### Transfection and proliferation assay

Cells underwent reverse transfection with 50 nM siRNA by using Lipofectamine RNAiMAX Transfection Reagent (Life Technologies) according to the manufacturer's instruction. For immunoblotting, cells were cultured in a 6-well plate. For the proliferation assay, cells were cultured in triplicate in a 96-well plate and incubated in IncuCyte (Essen BioScience, Ann Arbor, MI, USA). Proliferation of cells was monitored at 4-h intervals, and confluence (%) was calculated by IncuCyte software.

### Immunofluorescence, chromosome spreads, senescence assay and apoptosis assay

Indirect immunofluorescence and quantitation of kinetochore signals were performed as previously described.^38^ Chromosome spreads were achieved as previously described.^25^ Senescence assays were performed as previously described.^39^ Briefly, 10^4^ MEF cells were cultured in a six-well plate in triplicate for 4 days. Cells were fixed with 2% formaldehyde (Fisher Scientific, Pittsburgh, PA, USA) and 0.2% glutaraldehyde (Sigma-Aldrich) in phosphate-buffered saline at room temperature for 5 min. SA-β-gal activity was quantified by counting 200 cells per well. For apoptosis assays, 1.3 × 10^5^ MEF cells were cultured in a 6-well plate in duplicate for 2 days. Cells were harvested and stained with annexin V-FITC and propidium iodide by using the Annexin V-FITC Apoptosis Detection Kit (Abcam). FACS analysis was performed with the BD LSR II (BD Biosciences).

### Clinical samples

The tissues from patients with breast cancer, lung cancer, Ewing sarcoma and rhabdomyosarcoma were obtained from Cooperative Human Tissue Network, University of Pennsylvania, Philadelphia, PA, USA and Nationwide Children's Hospital.

### Immunohistochemistry of Sgt1

Immunohistochemistry study to determine the distribution of Sgt1/Sugt1 protein in tissue sections from patients with breast cancer, lung cancer, Ewing sarcoma and rhabdomyosarcoma were performed as described previously with a minor modification.^22^ Briefly, deparaffinized tissue sections were heat-retrieved in 0.01M citrate buffer and immunohistochemically stained with the primary rabbit polyclonal antibodies against Sgt1/Sugt1 (Protein Tech Group Inc., Chicago, IL, USA) at a dilution of 1:250–1:500. Detection reagents were MACH 2 rabbit HRP polymer (Biocare Medical, Concord, CA, USA) followed by Liquid DAB Substrate and Chromagen system (DAKO, Carpinteria, CA, USA). The sections were counterstained with Mayer's hematoxylin. The images of the stained tissues were acquired on a Zeiss AxioScope microscope system (Zeiss, Dublin, CA, USA) equipped with Plan-NEOFLUAR objective lenses and the AxioCam HRc high resolution digital camera (Zeiss) using AxioVision software (Zeiss).

## Supplementary Material

Suppelementary Information

Suppelementary Figures
