# Supplementary material for: The oncogenic role of the cochaperone Sgt1
Source: Oncogenesis. 2015 May 18;4(5):e149–. doi: 10.1038/oncsis.2015.12 (PMC4450263; doi:10.1038/oncsis.2015.12)
Supplement: Suppelementary Information [file oncsis201512x1.doc]

**Figure S1. Sgt1 Protein Is Overexpressed in Tumor Tissues.** (A-E) Ready-to-use membranes containing 14 μg of proteins of tumor tissues (T) and normal adjacent tissues (N) from the same patient were used to detect Sgt1 by immunoblotting. Grade, stage, sex, and age are shown.

**Figure S2. *Sgt1* Homozygous Knockout Mice Experience Early Embryonic Lethality.** (A) Schematic diagram of *Sgt1* knockout allele. The gene trap vector was inserted into intron 2. (SA, splice acceptor; b-geo, galactosidase/neomycin phosphotransferase fusion gene; pA, polyadenylation sequence) (B) *Sgt1* heterozygous knockout female and male mice were crossed. Genotype frequencies of the indicated progeny are shown. (C) Genotypes of E3.5 embryos produced by in vitro fertilization and of E8.5 to E14.5 embryos were determined. Genotype frequencies of the indicated embryo are shown.

**Figure S3. *Sgt1*^+/-^ MEF Cells Have Proper CENP-H Loading onto Kinetochores and a Distribution of Chromosomes Similar to That of *Sgt1*^+/+^ MEF Cells.** (A) Sgt1 and β-tubulin in *Sgt1*^+/+^ or *Sgt1*^+/-^ MEF cells were detected by immunoblotting. The Sgt1 protein level was normalized to that of β-tubulin, and the protein level in *Sgt1*^+/+^ MEF cells was established as a value of 1. (B) Indirect immunofluorescence of *Sgt1*^+/+^ and *Sgt1*^+/-^ MEF cells was performed. DAPI (blue), CENP-H (green), and CREST (red) were immunolabeled. CENP-H signals were normalized to CREST signals, and the signal of *Sgt1*^+/+^ MEF cells was established as 100%. Average values ± SD are shown. (C) Chromosomes in more than 90 MEF cells were counted. The distribution of chromosome number is shown.

**Figure S4. *Sgt1*^+/-^ *p53*^-/-^ MEF Cells Have a Phenotype Similar to That of *Sgt1*^+/+^ *p53*^-/-^ MEF Cells.** (A) The mitotic index of *Sgt1*^+/+^ *p53*^-/-^ and *Sgt1*^+/-^ *p53*^-/-^ MEF cells in response to treatment with 250 nM paclitaxel was determined. The mitotic index was calculated by counting MEF cells with phosphorylated histone H3 per 200 cells. Average values ± SD are shown. (B) Senescence of *Sgt1*^+/+^ *p53*^-/-^ and *Sgt1*^+/-^ *p53*^-/-^ MEF cells was measured by β-galactosidase assays. MEF cells were infected with pBABE-puro and pBABE-puro Hras retrovirus. Wild-type MEF cells were used as a positive control. Average values ± SD are shown. (C) Apoptosis of *Sgt1*^+/+^ *p53*^-/-^ and *Sgt1*^+/-^ *p53*^-/-^ MEF cells was measured by annexin V-FITC/PI staining followed by FACS analysis. Average values ± SD are shown. (D) Confirmation of Knockdown of *Sgt1* Expression. HCT 116 and MDA-MB-231 cells were transfected with *Luc*, *Sgt1-1*, and *Sgt1-2* siRNAs. Knockdown of *Sgt1* expression was confirmed after 72 h by immunoblotting.

**Figure S5. Immunohistochemistry.** Immunohistochemistry studies of formalin-fixed, paraffin-embedded surgical sections of breast carcinoma (A), normal breast tissue (B), lung carcinoma (C), normal lung tissue (D), Ewing sarcoma (E), normal cartilage (F), rhabdomyosarcoma (G) and normal cartilage (H) using an anti-Sgt1 antibody. The boxed region in the image at x20 magnification is zoomed and shown at x40 magnification.
